# P02.34. Therapeutic effects of traditional Chinese medicine in cancer patients undergoing chemotherapy or radiotherapy: randomized, double-blind controlled trial

**DOI:** 10.1186/1472-6882-12-S1-P90

**Published:** 2012-06-12

**Authors:** L Lo, C Chen, C Chang, T Lee, M Hou, T Cheng, J Chiang

**Affiliations:** 1 Changhua Christian Hospital, Changhua, Taiwan; 2 Department of Mathematics and Institute of Statistics and Information, Changhua, Taiwan; 3 Department of Computer Science and Engineering, National Sun Yat-sen, Kaohsung, Taiwan

## Purpose

Cancer is one of the most major health issues worldwide. An increasing number of cancer patients received surgery, chemotherapy and radiotherapy. However, there usually exists some side-effects. Traditional Chinese Medicine (TCM) is one of the most common complementary therapies used in Taiwan. We designed a randomized, double-blind and placebo-controlled clinical trial to evaluate the role of TCM on patients with cancer.

## Methods

Inclusion criteria were being diagnosed as cancer patients within 3 years and received chemotherapy (CT) or radiotherapy (RT), age above 18 years old. Exclusion criteria were pregnancy, brain metastasis, or receiving drugs for other clinical trials. These patients were separated to an intervention group (Sheng-Mai-san) and a placebo group for 4 weeks using a randomized, double-blind principle. EORTC QOL-C30 was used to evaluate quality of life. General data, CBC, GOT, GPT, BUN, creatinine, and CEA were also recorded. These data were collected at baseline and one month after using drugs. Enrolled patients were prescribed sealed granules, which contain therapeutic drugs or placebo. We used paired-t tests for statistical analysis.

## Results

Forty patients were recruited in this study. Twenty-nine completed the EORTC QOL C30 questionnaire and laboratory data. There were fourteen patients in the intervention group including twelve females and two males. Fifteen patients were in the placebo group, including nine females and six males. Patient characteristics of the two groups were similar. We compared the total scales of EORTC-C30 pretreatment and post treatment and there were significant differences in insomnia, constipation and financial difficulties in intervention group (p<0.05). Besides, we also noted significant decreases in WBC in the placebo group (p=0.01). But neutrophils increased significantly (p<0.05 ) and average WBC increased (still within normal limits) in the intervention group.

## Conclusion

TCM (Sheng-Mai-san) improved several symptoms in cancer patients undergoing CR or RT and prevented decreases of WBC. TCM may be a complementary therapy for cancer patients undergoing CT/RT.

